# Precise Coulometric Titrations of Halides

**DOI:** 10.6028/jres.067A.005

**Published:** 1963-02-01

**Authors:** George Marinenko, John K. Taylor

## Abstract

A method has been developed for the precise assay of halides by constant-current coulometric titration with silver ions generated at a silver anode. It is shown that a 4 milliequivalents sample of halide can be titrated with standard deviation of 0.005 percent.

## 1. Introduction

The determination of halides is of interest in many chemical investigations. For example, in a recent determination of the atomic weight of chlorine[1]^1^ it was necessary to determine the chloride content of dilute solutions with high precision. For such applications, coulometric titration is particularly advantageous because it involves a minimum of manipulation. A further advantage is that the titrant is generated in situ in amount that is measured in terms of physical quantities.

Although a number of publications have been concerned with the coulometric determination of halides [2, 3,4, 5] there seem to have been no attempts made to establish the conditions for high-precision titrations, i.e., better than 0.1 percent. Accordingly, the work reported in this paper was undertaken as part of a continuing program at the National Bureau of Standards to develop coulometric titrations of high precision and accuracy[6].

## 2. General Considerations

Coulometric generation of silver ion has been extensively studied. One of the more recent studies was done by D. N. Craig and coworkers [7, 8] in connection with the determination of the value of the faraday. The electrode reaction Ag→Ag^+^+e^−^ (or one equivalent thereto) proceeds at 100 percent current efficiency for current densities as high as 0.150 amp/cm^2^ [7]. In the work reported in the present paper, the total area of the silver anode was about 20 cm^2^ and the generation current was 100 ma; thus the current density did not exceed 0.005 amp/cm^2^. At such low current densities, one is assured of having 100 percent current efficiency with respect to the generation of silver ion provided no less noble metals are present in the metallic silver. For this reason spectrochemieally pure mint silver (stated purity, 99.999%) was utilized for constructing the generator anode. Nonoxidizable impurities in silver such as silica do not present any problems since the amount of silver ion produced by the electrochemical reaction is important rather than the weight loss of the anode. Accordingly, no need for further purification of silver was warranted at this time, since other uncertainties of the method were more significant.

All calculations were based on the 1961, C^12^ atomic weight scale. The molecular weights used for NaCl, NaBr, and KI were 58.442_8_, 102.898_8_, and 166.006_4_, respectively.

The value of the faraday constant used was based on the determination of D. N. Craig et al. [7]. Using their electrochemical equivalent of silver, 1.117972 mg/coul, and the atomic weight of silver 107.870 g/mole on the new C^12^ scale, the value of the faraday is 96,487.2 coul/g-equiv.

## 3. Apparatus and Procedure

### 3.1. Apparatus

The design of the titration cell in this work was similar to that described in the previous communication of this series [6] except that the new cell was of smaller capacity. The anode and the cathode compartments were 180 ml electrolytic beakers connected horizontally by a 25 mm o.d. tube, approximately 10 cm in length, in which a coarse, a medium, and a fine porosity sintered glass disk were sealed. Each of the two intermediate compartments thus formed had a tube with a stopcock to enable filling and emptying, by applying vacuum or nitrogen pressure.

The anode was a heavy piece of silver 3 cm×3 cm and about ½ cm thick, made by melting together a number of thinner sheets of mint silver by placing them in a “sandwich” on a heavy plate of silver and applying heat with an oxygen torch. To permit mounting in the cell cover and to facilitate connection of the lead wire, the anode was attached to a 12 cm long cylindrical silver handle, 1 cm in diameter, fabricated from the same material. The anode thus formed was etched in dilute HN0_3_ (1:10) to remove any surface impurities, and washed with large quantities of distilled water.

A piece of corrugated platinum foil (5 cm×16 cm) served as the cathode in the generation circuit. The large surface area thus obtained was necessary to minimize polarization of the cathode and to decrease current fluctuations due to variation in cell resistance as a consequence of the liberation of hydrogen gas.

The electrical circuit used in this work was the same as described in the previous communication [6].

The generation current for all titrations was 101 ma and its exact value was determined by the value of the standard resistor and the voltage of the saturated Weston cell. Any small initial unbalance between the *IR* drop across the standard resistor and the standard cell was corrected quickly after which the *IR* drop across the standard resistor was maintained equal to the voltage of the Weston Cell. The value of the resistor used was 9.99994 ohms at 25.0 °C and its temperature was maintained constant in a thermostated oil bath. Nevertheless, the actual temperature of this resistor was measured before and after each titration with a mercurial thermometer, and corresponding corrections were applied, based on the mean value of temperature, by the use of the relation *R_t_*=*R*_25_[1+*α*(*t*−25)+*β*(*t*−25)^2^] ohms.

In the course of a titration, the temperature of the standard resistor increased from the 25 °C bath temperature by as much as 1 °C. However, the correction for this difference amounted to only 7 ppm. Since the measurements of the temperature were made to a tenth of a degree the mean value of the resistor during the run was known to better than 1 ppm. The value of the saturated standard Weston cell used for the comparison of the *IR* drop of the standard resistor was 1.017875 v ± 0.8 *μ*v at 34.099 °C. The temperature of the Weston cell was always maintained at that value in a specially built thermostated box.

Near the equivalence point of a titration, the current was passed incrementwise. It was found advantageous to use a commercially available constant-current coulometric titrator for this purpose, because the size of these increments can be conveniently and adequately controlled and reproduced using this instrument without any appreciable sacrifice of accuracy. The timing circuit and current of this instrument were calibrated and the *It*-product was found to be accurate to ±0.005 percent.

As in the case of acid and base titrations, it was found that a gel plug was necessary to eliminate the flow of electrolyte between the anode and the cathode compartments. In this work a silicic acid gel plug was used. It was conveniently prepared by mixing together 10 ml of 1:4 water diluted solution of technical grade sodium silicate (40 Be) with 5 ml of 1.0 *M* HN0_3_. The resulting solution was poured onto the frit of the cathode compartment. Gelation of this solution occurred in approximately 2 min after initial mixing.

The amperometric indicator circuit consisted of two silver electrodes and a polarograph. The indicator cathode was a silver bead approximately 1 cm in diameter (3 cm^2^ in area); the indicator anode was a bead approximately 0.5 cm in diameter (0.8 cm^2^ in area). Both beads were made by melting the same silver as used for fabrication of the generator anode. These in turn were fused to pieces of platinum wire and the latter sealed into glass tubes to facilitate electrical contact. The polarograph was used as a source of the applied emf between these two electrodes and also as a recording microammeter, to measure the indicator current. The applied emf was 0.050 v.

All weighings were done on a 20-g capacity microbalance and were precise and accurate to ± 0.003 mg. All weighings were corrected for air buoyancy.

### 3.2. General Procedure

The preparation of the coulometric cell consisted of thorough cleaning with fuming sulfuric acid followed by passage of large quantities of distilled water through the glass frits. Subsequently the cell was exposed to prolonged steam cleaning by forcing the steam to pass through the frits, followed again by several rinses with distilled water.

After such cleaning a silicic acid gel plug was poured onto the fine porosity frit of the cathode compartment.

The cell was surrounded by aluminum foil to shield the contents of the cell from light. About 100 ml of 0.1 *M* HNO_3_ saturated with NaNO_3_ were poured into the cathode compartment to which about 5 *μ*eq of AgNO_3_ solution were added to precipitate traces of halides or other impurities which form insoluble precipitates with silver. Otherwise, these could cause positive errors by migration into the anode compartment. A slight excess of silver ion would not cause any negative errors since the electrical migration forces would keep it from diffusing into the adjacent compartment.

For each titration 100 ml of anolyte were used. The anolyte was 1 *M* NaNO_3_, 1 *M* acetic acid in 50 percent methanol. The methanol was added to suppress the solubility of silver halides [2].

The platinum cathode was placed in the cathode compartment and a Teflon-encapsulated magnetic stirring bar was lowered into the anode compartment by means of another magnet.

A polyethylene cover, supporting the generator anode and two indicator electrodes, was then secured on the anode compartment by means of a snugly fitting lock ring.

At this point the cell was placed on the platform of a magnetic stirrer, operated from a constant voltage transformer to insure constant stirring rate which is imperative for precise amperometric end-point detection.

Sufficient anolyte was permitted to flow into the intermediate compartments to just wet the bottom of each of the intermediate compartments, thus establishing electrical contact. About 10 *μ*eq of halide were introduced into the anode compartment, through an opening in the cover, to facilitate pretitration.

At this point, a 6.43 ma current was passed incrementwise (1 *μ*eq increments) with the coulometric analyzer. The indicator current was recorded after the passage of each increment. This pretitration was continued until the excess of silver ion was about 10 *μ*eq or in terms of concentration 10^−4^*M*. The portion of the indicator current line, after the equivalence point, where the indicator current becomes a straight line function of the silver ion concentration, was extrapolated to zero current which was chosen to be the end point of both the pretitration and the final titration.

After the pretitration was completed, the intermediate compartments were completely filled with anolyte and the silver ion was generated using the high-precision manually controlled constant-current circuit. The amount of silver which was generated at this point was a precalculated quantity, sufficient to react with about 99.5 percent of the halide expected to be found in a given sample.

Following the generation of silver ion the sample was introduced into the anode compartment, and the intermediate compartments were emptied by pressure until only the bottoms of these compartments were slightly wetted by the solution. At this point the titration was continued, using again the coulometric analyzer, until the indicator current passed a minimum and began to rise as the concentration of silver ion was increased. The titration was again stopped and the intermediate compartments were subjected to several successive rinsings with anolyte. From this point on, the titration was continued incrementwise in the same fashion as the pretitration. The amount of electrogenerated silver ion equivalent to the halide present is obviously the amount generated to the end-point plus the excess generated in the pretitration step.

It was found, in the preliminary part of the work, that in the case of large samples, e.g., 4 meq, erratic results were obtained if the sample was introduced into the cell before generation of silver ion, in that the generation current was very difficult to maintain constant after the generation of the first milli-equivalent of silver ion. This phenomenon was caused by the dense precipitate deposited directly on the silver anode thus hindering the diffusion of the generated silver ion into the bulk of solution. This problem was solved by the above described procedure, that is, by pregeneration of the major portion of silver ion before the introduction of the halide sample into the cell. The amount of halide which still remained to be titrated after the introduction of sample was so small that the interference due to coating over of the electrode by the precipitate was eliminated.

Another problem which arose as a result of the pregeneration of silver ion was the delivery of sample. It was found that a solid sample could not be introduced directly into the silver ion solution because the halide crystals became instantaneously encapsulated by a film of silver halide precipitate and again inclusion errors were inevitable. Accordingly, the samples were either weighed into the cell in the form of solution or, in the case of solid-weighed samples, they were dissolved prior to delivery into the cell.

## 4. Titration of Sodium Chloride

### 4.1. Procedure

The sodium chloride which was used in this work was one purified by double recrystallization of a reagent grade salt by precipitation with HCl-gas from saturated solution and subsequent fusion in a platinum crucible. The large crystals of NaCl thus obtained were crushed in an agate mortar and dried at 110 °C. This material was stored in a desiccator until used. The spectrochemical analysis of this material showed the total metallic impurities did not exceed 0.004 percent.

Two sets of experiments were performed with sodium chloride, one involving the preparation of solutions which were later dispensed into the cell from polyethylene weighing bottles, and the second set involving weighing of sodium chloride as solid.

The solutions were prepared to be of such concentrations that the desired amount of chloride (1 meq or 4 meq) was contained in approximately 10 g of solution. The following were the concentrations of the two solutions on a vacuum-corrected basis: Solution A—0.1031324 meq/g; solution B—0.4202323 meq/g. The actual weight of a sample was obtained by difference in weight of the weighing bottle before and after the delivery of a sample.

Solid samples were weighed in a small boat also by the difference method. The crystals were delivered into a funnel, containing a Pyrex wool plug and leading into the anode compartment. After the generation of 99.5 percent of the silver ion required, as described above, the sample was washed down with 10 ml of distilled water. Near the end point the funnel was rinsed a number of times with anolyte withdrawn from the cell by means of a syringe to which a glass tube was attached in place of the customary needle.

### 4.2. Results

The results obtained for a number of titrations over a period of several months are given in the table 1, columns 2, 3, 4, and 5. The agreement among these sets is remarkably good and well within the limits of standard deviation for each set. The precision for each of the sets is much better than could be obtained on the same size samples by conventional gravimetric or titrimetric methods.

**Figure 1:**
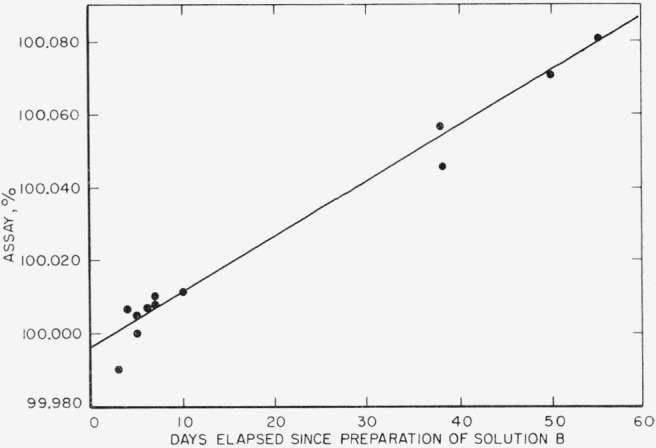
Change of assay of solution B with time.

It is apparent from the examination of the data that solution B of sodium chloride was increasing in concentration with time (fig. 1) even though both solutions were kept tightly closed in the ground glass stoppered bottles in water-vapor saturated bell-jars. In the case of solution A, this effect is not as pronounced as in the case of solution B. Two reasons account for this behavior. Firstly, the volume of solution A was 1,000 ml, while the volume of B was 200 ml. Thus for a given amount of water lost, the concentration change would be amplified by the factor of 5 in solution B over that of solution A. Secondly, the standard deviation for 1 meq samples is four times larger than for 4 meq samples; thus a trend in concentration change would be masked more in the case of solution A.

An equation of the form *A*=*A*_o_+*bt* was fitted to the data of solution B by the method of least squares to express the assay of solution at any time since the time of preparation. In this equation *A* is assay at any time, *t*, *A*_o_ is the assay of this solution at the time of preparation, *b* is the slope of this line expressed in precent day^−1^ and *t* is time elapsed since the preparation of solution in days. The solution of the above equation yields 0.00150 percent-day^−1^ for the slope *b* of this line, and 99.996 percent for *A*_o_ as indicated at the bottom of column 3, table 1. This value for the assay is in excellent agreement with both the expected value and the assays of the solid weighed samples of the same material.

## 5. Titration of Sodium Bromide

### 5.1. Procedure

The sodium bromide used for these experiments was a reagent grade salt. It was dried at 110° C and stored in a desiccator for use without any preliminary purification. The samples were weighed out as solid NaBr in the same manner as the solid NaCl samples.

### 5.2. Results

Two sample sizes, 1 meq and 4 meq, were investigated as in the case of NaCl and the results are given in columns 6 and 7 of table 1. The average value for the assay in both cases is in excellent agreement. The value of the assay appears to be 0.17 percent too high and undoubtedly is caused by the presence of chloride impurity since the amount of chloride in this salt is known to be of the order of 0.2 percent by weight. The standard deviations for both the larger and the smaller samples are of the same order of magnitude as in the case of sodium chloride, 0.038 percent for 1 meq and 0.009 percent for 4 meq samples.

## 6. Titration of Potassium Iodide

### 6.1. Procedure

The potassium iodide was a reagent grade salt which was not subjected to any further purification. It was dried at 110 °C and stored in a desiccator before weighing. The choice of the potassium salt over sodium was made because sodium iodide is a very strongly hygroscopic material, which impairs accurate weighings.

In the vicinity of the end point of iodide titrations, the indicator current exhibited three inflection points, probably due to strong adsorption of the silver ion after the concentration of the latter reaches certain critical values. This, of course, indicated that several equilibria are to be satisfied, which brings in an uncertainty, or at least a doubt in the experimenter’s mind as to the time to allow for equilibrium to establish. The problem was circumvented by the addition of 1 ml of 0.01 *M* solution of NaBr just as this end-point phenomenon occurred. The solution which was added was standardized previously by the coulometric technique and the number of milliequivalents in a 1-ml aliquot were subtracted from the total number of milliequivalents found in the sample. By this technique one in fact is determining the iodide by observing the bromide end point. In order to make the end point of the titration equivalent to the end point of the pretitration, a small amount of the same NaBr solution was added before the pretitration.

### 6.2. Results

The results for titration of 1 and 4 meq samples of potassium iodide are shown in columns 8 and 9 of table 1. Here again the precision for each set is of the same order of magnitude as in the case of the other two halides. The standard deviations for 1 and 4 meq samples are 0.030 and 0.007 percent respectively. The mean value of the assay is larger for larger samples which can he caused by the adsorption of silver ions on the silver iodide precipitate.

## 7. Discussion

This work, similarly to the previous communication of the acidimetric titrations, shows the precise nature of coulometric titrations, with the attractive feature of utilizing a titrant the amount of which is based upon physical measurements rather than chemical standards of reference.

Needless to say, the experimenter must exercise extreme control over many factors such as weighing, delivery of sample, preparation of sample, current control, i.e., all factors which affect the precision and accuracy of any chemical analysis.

One factor which is especially important in these titrations and may very well apply to other precipitation titrations with amperometric end point detection, is the interference of the precipitate with the indicator electrode system. It was found that with large samples, the heavy precipitate which is formed may accumulate on the indicator electrodes, and in several instances it was found to form a bridge between the indicator anode and the indicator cathode. Such occurrence impairs the sensitivity of the indicator system by lowering the slope of the indicator current line. This condition, however, was simply eliminated by tapping the indicator electrodes before taking the final indicator current readings, to loosen the adhering precipitate.

**Figure 2:**
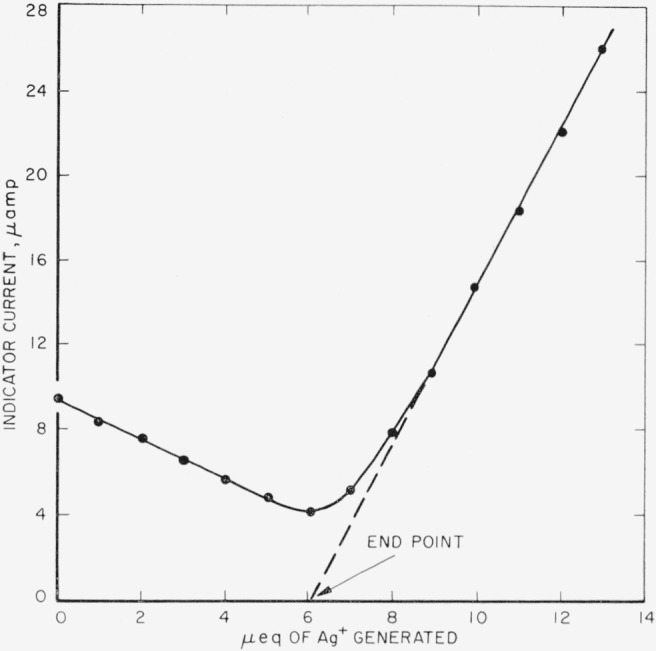
Typical end-point determination.

At no time did the indicator current curves resemble those described by Lingane [9], even though the applied emf across the two indicator electrodes was 50 mv as he suggested. In all cases the indicator current was high before the end point, decreased as the end point was approached, passed through a minimum in the vicinity of the end point, and became a straight line function of the generated silver ion after the end point (fig. 2).

In view of the fact that a potential difference of only 50 mv was applied across indicator electrode system, it is very difficult to believe that a high indicator current before the end point was caused by the reduction of hydrogen ion at the indicator cathode. It may be explained by the reduction of the colloidal silver halides or silver halide complexes, since it is well known that silver chloride is appreciably soluble in high concentrations of chloride ion.

The pretitration procedure was devised to minimize uncertainty in the end-point location due to the absence of a well-defined residual current. By this technique the silver-halide equivalency is obtained from the difference between the end points, similarly determined, so that errors tend to cancel.

## 8. Conclusions

It has been shown that chlorides, bromides, and iodides can be titrated with high degrees of precision by coulometrically generated silver ion. In the case of sodium chloride, it has been shown that this method is accurate as well as precise. In the course of this work it has also been shown that it is rather difficult to maintain solutions of constant concentration. In working with solutions one is obliged to determine the rate at which the solution composition is changing to permit extrapolation of composition— time equation to time=0.

## Figures and Tables

**Table 1:** Summary of results

Determination number	NaCl solution A	NaCl solution B	NaCl weighed solid		NaBr weighed solid		KI weighed solid	
	1 meq assay	4 meq assay	1 meq assay	4 meq assay	1 meq assay	4 meq assay	1 meq assay	4 meq assay
								
1	99.970	99.990	100.018	100.003	100.156	100.172	99.910	99.972
2	99.979	100.007	100.012	100.016	100.122	100.163	99.905	99.960
3	99.997	100.005	99.954	99.994	100.187	100.170	99.923	99.960
4	100.026	100.000	99.990	99.997	100.226	100.177	99.976	99.975
5	100.029	100.007	99.943	100.000	100.133	100.188	99.881	99.976
6	100.022	100.008	99.974	100.001	100.191	100.169	99.921	99.965
7	99.999	100.010	99.983	………	100.133	………	99.940	………
8	100.016	100.011	100.013	………	100.142	………	………	………
9	99.994	100.056	………	………	100.211	………	………	………
10	………	100.045	………	………	100.208	………	………	………
11	………	100.070	………	………	………	………	………	………
12	………	100.081	………	………	………	………	………	………
Mean	100.004	a99.996	99.986	100.002	100.171	100.173	99.922	99.968
Standard deviation	0.021	0.005	0.028	0.008	0.038	0.009	0.030	0.007

a The mean value of the assay corrected for the change in concentration of the solution with time.

## Footnotes

1 Figures in brackets indicate the literature references at the end of this paper.
